# Calmeil on Mental Epidemics

**Published:** 1849-04-01

**Authors:** 


Art. II.—*De la Folie, considerée sous le point de vue pathologique, philosophique, historique, et judiciaire depuis la Renaissance des Sciences en Europe jusqu'au Dix-neuvième Siècle ; description des grandes Epidemies de delire simple ou compliqué, qui ont atteint les populations d'autrefois, et regné dans les monastères ; exposé des condamnations auxquelles la folie méconnue a souvent donné lieu.* Par L. F. Calmeil, Docteur en Médécine de la Faculté de Paris, Médécin de la Maison des Aliénés de Charenton, Membre de la Legion d'Honneur. Paris. 2 Tom.

*On Insanity, considered in a pathological, philosophical, historical, and judicial point of view, from the Revival of the Sciences in Europe to the Nineteenth Century ; a description of the great epidemics, of simple or complicated insanity, which attacked the people in former times, and prevailed in the monasteries ; with an exposé of the judgments which unrecognised insanity has led to.* By L. F. Calmeil, M.D., Physician to the Asylum at Charenton, and Member of the Legion of Honour. Paris. 2 vols.

There are few studies so interesting as the history of the great epidemics which have afflicted the human mind. The scourges by which the body is diseased, and whole regions of the earth laid waste and desolate : plague, cholera, yellow fever, black death, and sweating sickness have all their respective points of interest, and deservedly engage the attention of the philosophical physician. Their causes, characters, and effects have been as fully investigated as the progress of science has hitherto permitted. Of the works of this character which treat generally of epidemics, that of Hecker, on "*The Epidemics of the Middle Ages,*" an English version of which has been published by the Sydenham Society, is perhaps the best and the most interesting. The disordered and diseased conditions of the human mind, when occurring widely among the population, and leading to extraordinary manifestations and aberrations of intellect and conduct, although generally noticed by medical writers, have not hitherto been treated of historically and philosophically. M. Calmeil, than whom, no one was better fitted to be an historian, has assumed the Herculean task, and performed it with an energy and skill, which must add greatly to his reputation. Not content with describing the epidemics, and displaying their prominent features, he has traced out their connexion with disordered conditions of the mind, and has shown that persons who were burnt, or otherwise executed for magic and other offences, ought instead, if psychological science had been sufficiently advanced, to have been sent to lunatic asylums. We may rejoice that we live at a time when the progress of science has stripped off the veil that enshrouded and obscured the wanderings of the mind, which led the great mass of mankind to consider their signs as indications of dealings with the fiend, instead of, as they really were, the manifestations of disordered intellect, perverted by the condition of society, —the overwhelming influence of a bigoted, narrow-minded priesthood, and the darkness of general ignorance. The insane in the present time, no matter whether the hallucination refer to this or to the invisible world—no matter whether the lunatic conceive himself to be Cæsar or Christ—stands in no danger of execution as a political or religious offender. He stands aloof alike from the scaffold and the stake, and the care of those who have him under control is directed to clear and restore a disordered intellect, and prevent his becoming an obstacle to the well-being of society, and not to render him amenable to its laws. A few centuries ago, Irving would have perished at the stake as a religious impostor. Thom, had he not been slain by the soldiery, would have died on the scaffold or at the stake, on the same pretence. Their insanity would not have been recognised. We manage these things better now.

The first book of the work before us, which consists of one chapter only, treats of "Insanity, its functional elements, and its principal modes of manifestation in the simple and complicated states." This is simply introductory, and need not detain us long. In it are described the hallucinations of the different senses to which maniacs are subjected, and their connexion in a great many cases with existing morbid changes in the organs to which they are referred, explained. The different causes and characters of insanity, together with their origin in the perverted passions and feelings of humanity, also occupy a considerable portion of this chapter. Great stress is laid on the super-excitement of the organs of generation as a frequent cause of insanity, evinced in the first instance by the extreme indulgence in the passions connected with the functions of these organs, such as inordinate, misplaced, or ill-regulated love, jealousy, &c. Numerous cases are narrated, illustrative of this point. M. Calmeil thus sums up the effects of hallucinations &c.:—

"The hallucinations, false sensations, erroneous ideas, false judgments, the alienation of the moral faculties, the disorder of the will, are among the principal elements of insanity. These functional lesions sometimes succeed each other in the brain without any apparent order, and as it were by chance; at others, they follow each other with an incontestable regularity and sequence. We may sometimes observe an almost logical affiliation between the diseased sensations, the delirious ideas, and the different lesions of the instinctive and moral faculties; in other cases, the connexion between the different functional disorders does not appear precisely necessary; but, as in a state of reason, an idea, a recollection, an emotion is awakened under the influence of such or such a sensation, rather than under the influence of such or such another, so, in delirium, the existence of certain morbid phenomena is often connected with that of such or such a lesion, in preference to that of such or such another. In the different species of monomania, we are able sometimes to ascertain whether the derangement of the psychical faculties has commenced by an absurd idea, by an illusion of the senses, by an hallucination of the smell, sight, taste, hearing, or touch, by a vicious super-excitement of the passions, by the alienation of some feeling; in these diseases, we can equally be certain that not only the primitive alienation leads to a certain number of others, but also that there often exists a natural analogy between the phenomena which have been noticed above, at the commencement, and those which have afterwards aggravated the condition of the patient."

The concluding part of the chapter treats of idiocy, imbecility, the complications of insanity, mania, monomania, partial delirium, theomania, demonolatria, demonopathia, and zoanthropia—all forms of insanity which have more or less prevailed in the world, and which, in the antecedent centuries, raged epidemically, and led to the exercise of the most revolting cruelties on their unhappy victims. M. Calmeil's object in writing this preliminary sketch of the various disorders and diseases affecting the human mind, has been to prepare the reader, and enable him duly to appreciate the evidences of insanity brought forward when narrating the histories of the great epidemics of insanity which comprise the greater part of this valuable work. His account of hallucinations, and of the characters of insanity, is exceedingly well written, and largely illustrated by a series of instructive cases, evidently bearing on every part of the question. True, they are isolated instances, but they are not on that account the less valuable evidences in favour of the opinion that epidemic diseases, showing similar features, may be traced to the same origin, in the same way that an attack of intermittent fever occurring in London, will differ in intensity from the same disease met with epidemically in marshy districts.

The second book is devoted to the consideration of insanity in the fifteenth century. It consists of two chapters, the first of which treats of theories, and the second of the theomania of Jeanne d'Arc, homicidal monomania, and pretended anthropophagia in the Vaudois, the demonolatria of Dr. Edeline, pretended anthropophagia in Germany, and the demonopathia of the nuns of Cambrai.

It was not to be expected that, after the long reign of ignorance and barbarism, the minds of men should be in a fit state to grapple with the difficulties attendant on the solution of the question respecting diseases of the intellect. "It is evident that the explanation of diseases of the understanding, of all the functional aberrations connected with the nervous apparatus, is based on the *ensemble* of anatomico-physiological, philosophical, and pathological data, which can be rendered available only by the effects of a slow and successive observation; and besides, mental pathology could not be at once freed from the chains with which it had been loaded in the high regions of metaphysics." Everything tended in these days to direct the attention of all classes of society to the existence in the world, and the direct communication of supernatural beings with the visible inhabitants of this earth. The idea that active and intelligent beings placed between God and man, between heaven and earth, were indispensable as occasional causes, then occupied the first place in the convictions of Christian doctors and metaphysicians. The consequence was that monomaniacs were generally classed as heretics, the disciples of Satan, and apostates. The Holy Scriptures, and the oral and written traditions of the church, led men to conclude in the still existing communications of angels and demons with men, and events which would now be explained as the results of natural causes, or as effects of disease, were then universally regarded as evidences of demonolatria, and punished rigorously and fearfully, as proofs of a falling off from the faith.

Insanity is apt to be tinctured by the religious belief, the philosophical or superstitious ideas, and social prejudices which are prevalent at the time among the people or nations; this varies even in the same country, according to the character of political events, civil commotions, the nature of literary productions, theoretical representations, and also according to the bearing of industry, the arts and sciences. The progress of science has caused many persons in their hallucinations, to refer their feelings to the influence of electricity, balloons, burning glasses, the telegraph, air guns, and to the effects of optical instruments. Many, since the opinions of Mesmer were first broached, considered themselves persecuted by magnetizers and somnambulists. Thus, in the fifteenth century, insanity bore the impression of the superstitious ideas and theological doctrines then in vogue, nor could it be otherwise, for these doctrines had been brought forward and developed in the schools, taught in the religious houses, explained to all the world from the pulpit, and amply commented on to the faithful when in confession. The hallucinations and false ideas of the insane had reference principally to demons, angels, and supernatural beings, precisely because they were familiar with these subjects, and because they had made a deep impression on their imagination. They were thus led to consider themselves as contemners of the true God, and apostles of the fiend. The atrocities of the inquisition greatly increased this tendency, many being forced by torture to make such confessions, who became afterwards victims at the stake. Thus ignorance and fanaticism, as they ever do, conduced greatly to the spread of insanity.

The insanity of Jeanne d'Arc is regarded by our author as an instance of theomania, which acted by influencing her warlike ardour, by communicating to her an extraordinary air of command, and a great excitement (*illumination*) of the understanding, rather than by falsifying the combination of the mind, and the rectitude of her judgment. Jeanne was early noticed by her desire for contemplation and melancholy. She rarely mingled in the sports of her companions, and when she culled flowers, it was to adorn the image of the Virgin, or of some other saint. The management of horses and hard labour afforded her pleasure. The recital of battles, and the woes of her country, which constantly formed the topic of conversation among the villagers, moved her greatly; frequent visions and secret ecstasies, favoured, doubtless, by the absence of menstruation, seemed to fix her destiny. Frequent hallucinations of sight and hearing commenced from her 13th year; when older, she believed that St. Michael, Gabriel, St. Catherine, and St. Margaret, visited her, and urged her on to the course she afterwards adopted. In these statements she ever afterwards persisted. It is, of course, certain that these personages never presented themselves in reality to Jeanne; like all other hallucinates, she was the dupe of the state of fascination of her senses and her brain. It happened, however, that in considering the errors of her imagination and judgment as heavenly visions, she saved France, and gained immortal reputation. The success that attended her, however, is no proof that she was right in attaching faith to her visions. M. Calmeil in no respect attempts to justify the cruelty that condemned her to the stake; but he admits that, had she lived in private life, a similar fate, in those days, would have awaited her, and the judges would have condemned her to the stake, after hearing her avowals, and having heard the wonders she had executed after having predicted them. Her fate, therefore, was an evidence of the ignorance and credulity of the times.

The theomania under which Jeanne laboured, threatened, after her death, to become contagious among her sex. Two young girls living near Paris, declared themselves her successors in her mission. One, who persisted in her declarations after having been examined by an ecclesiastical commission, was burnt; the other, having been assured she was under the influence of the fiend, showed signs of repentance and escaped. A third Amazon, of a bad reputation, appeared at Metz, and supported the views of the competitors for the bishopric of Treves. The inquisitor Henry summoned her before his tribunal; but she made her escape, and afterwards married a French officer. Her appearance at Metz gave rise to a report that Jeanne was not burnt in reality, but allowed to escape by the generosity of the English.

A few years after the execution of Jeanne d'Arc, a rumour spread through the Pays de Vaud, that the environs of Berne and Lausanne were filled with sorcerers and cannibals. It was reported that thirteen victims had disappeared, who had been devoured. The authorities made inquiries into the matter; several persons were submitted to the torture, and a great many were burnt. The confessions of the unhappy wretches in question would apparently justify the inquisitors in their proceedings, were it not evident that these statements were the ravings of insanity, and not the description of actual occurrences. One of the women accused, and who was put to death at Berne, acknowledged that she belonged to the sect of devil-worshippers, and that unbaptized children, and even those who had been baptized, but over whom the sign of the cross had not been made, became the victims of their sorceries. Newly-born children were caused by their invocations to perish in their cradles, and the bodies, after interment, were dug up and boiled by the witches, until the flesh became liquid and potable. The remaining solid parts were formed into an ointment, which, when a sorcerer was anointed with it, conveyed him through the air, wherever he desired to go; the liquid that resulted from the process was given to the novices to drink; a few drops would initiate them into the secrets of the art, and render them as learned as the masters. These strange avowals were confirmed by others, and maintained even at the stake. Stadelein, who was regarded as a magician of the first class, and boasted that by pronouncing certain words, and observing certain processes, he could compel the fiend to send his subordinates on earth, and could cause thunder and hail to fall on the farms of others, added further, that he had caused seven infants to perish in the bosom of one mother, and had also contrived that for many years all the females in the house of the same woman should abort; this he had effected by a spell made of a lizard's body.

It is certain that, plunged in ignorance and superstition, as all nations were in the fifteenth century, influenced by a firm belief in the existence of magic, and a mundane intercourse of demons, with confessions so full and ample, and so strongly confirmatory of all they had conceived or heard respecting the alleged science, the judicial and ecclesiastical authorities could not do otherwise than condemn the unhappy maniacs to the flames as apostates, murderers, and devil-worshippers. The progress of science, and the increase of education, have given a different impulse to the perverted ideas and hallucinations of the insane; but if they were to recur now in the same form and character as among the Vaudois in the fifteenth century, the judicial treatment would be very different. With our author we should recognise that the infanticides, murders, cannibal feasts, and all the atrocities with which the unfortunate wretches, who were called sorcerers, were charged, had no other origin than their imagination, and that in all probability no one had seriously thought of establishing devil-worship anywhere. A monomaniac alone, when her life was in danger, would persist in affirming that her comrades and herself caused infants to perish by uttering certain words; that human fat gave those, who were anointed with it, the power of flying through the air; and that the juice of infants, when drank in small quantities, changed neophytes into illuminati. A madman alone could persuade himself that he caused cows and sheep to abort, that he could cause the death of infants even in the mother's bosom, and that he could compel the evil spirits to let loose the elements, and to destroy the harvests. Even now patients labouring under melancholia in our asylums accuse themselves of most atrocious crimes. They have defaced, robbed, and pillaged their benefactors; have had recourse to fire and poison, and have caused inundations, earthquakes, and epidemic diseases. It follows not, therefore, because the crimes which the Vaudois monomaniacs acknowledged were of a most awful and atrocious character, that they really obeyed the most frightful impulses, destroyed infants, and proceeded by the decomposition of their corpses to obtain their disgusting draughts. Cases are on record certainly in which monomaniacs have actually murdered children, and one, a female in Milan, who was broken on the wheel, followed up the murder by an act of cannibalism. But in these cases the bodies of the victims and the blood proved the commission of the offence; but such evidence was not obtained in Berne in any case, nor was any one of the accused caught *in flagrante delicto*, which led the theologians to infer that the demons aided them in the commission of these crimes.

The origin of the general belief of mankind in the murderous and cannibal propensities of sorcerers, is referred by M. Calmeil, to a Jewish tradition respecting a sorceress named Lilith, said to be Adam's first wife, whose principal occupation seems to have been the destruction of the Jewish infants. A sect of Lilithites is reported to have sprung up afterwards among the Jews. Among the Greeks, Lamia, a daughter of Neptune, founded a similar sect or sects, the duties consisting in destroying infants, travellers, and young men. Among the Romans, aged sorcerers were represented, disguised as striges, or owls, and, thus changed, entering the houses and sucking the life-blood of children, in the hope, it is alleged, of regaining their youth. These fables were fully credited by the inquisitors, who, in their writings, designated their victims as Lamias, malefactors, striges, and lestrigones. Is it to be wondered at, then, that the unhappy wretches who had repeatedly listened to the reproaches levelled elsewhere against the adorers of Satan, and the denunciations of their crimes, should, when they believed they had themselves become sorcerers, acknowledge the most horrible offences, and the judges condemn them to the flames in perfect security of conscience? The habit of cannibalism, therefore, among the nations of Europe, must be regarded as a fiction. Similar, and even worse, crimes, were, in the early ages of Christianity, alleged against its professors by the Pagans.

Edelin, or Edeline, a doctor of the Sorbonne, first drew attention to himself by preaching openly against the belief in the worship of the devil, which he denounced as imaginary; and he preached also the cruelty of putting persons to death who were merely visited by illusions of the senses. His eloquence silenced his opponents, and for a while arrested the effusion of blood. He himself, however, fell under the charges of the theologians, and was arrested. The replies which he made to the interrogations addressed to him show that he had become insane. He acknowledged, it would appear, that he had long been a devil-worshipper; had been transported by the fiend to the assemblies of the sorcerers, and had only obeyed his master in preaching that fiend-worship was imaginary. His sentence was perpetual imprisonment.

In 1459, charges of sorcery were brought very largely against the inhabitants of the Artois, many of whom confessed the crime, and implicated others as equally engaged in the commission of the offence. These latter, by the contrivance of the judges, were generally selected from the better classes of inhabitants, who, after suffering repeated tortures, were compelled to confess, and then allowed to purchase their lives by sacrificing their wealth. The lower orders, after confession, were burnt. In some instances the constancy and courage of some of the inhabitants were such that they resisted the tortures, and did not confess; and others again bribed the judges and witnesses to obtain an acquittal. The demonolatria attributed to the inhabitants of Artois may be regarded as the type of a moral contagion, which soon after presented the most extraordinary forms. The author attributes the wild and extravagant ideas of these insane persons to conceptions formed during sleep, which led them to believe and confess that they had left their homes, had been present at fiendish assemblies, had eaten and drank there, and perceived in the crowds innocent persons whose lives they endangered by their charges. It is evident that all those who, in accusing themselves or others, believed they were speaking the truth, had become insane, since they were unable to distinguish the false from the true. They were, nevertheless, regarded by the theologians as heretics and apostates.

Towards the close of the century, similar proceedings took place at Cologne, Mayence, Treves, Saltzburgh, and Breme; and Innocent VIII. issued a bull denouncing the demon-worshippers. The crimes alleged against them were of a similar character to those already described, and the punishments were proportionate. Forty-eight women were burnt to death in one place on the charge of infanticide, and connexion with incubi; the latter offence in itself a hallucination, and leading materially to the belief that the crime of child-murder was also an error of the imagination. Forty-eight more were burned at Constance and Ravensburg, who had made similar confessions. Humanity revolts against such fearful destruction of the insane. M. Calmeil refers these confessions to disordered visceral sensations and erroneous sensations of touch. Midwives, who were also sorcerers, were especially regarded as guilty of infanticide; and it is said that the devil neglected nothing to get them into his service, on account of the opportunities they had of destroying newly-born children. One of them confessed to having killed more than forty children. Notwithstanding monomania may be homicidal, M. Calmeil is unwilling to believe that these women could have been guilty of the crimes they confessed, as that would involve the persistence of the homicidal monomania for years, without the existence of such a state being discovered by any one up to the moment of their arrest, and also that they must have destroyed hundreds of infants without the cognizance of their parents, a thing he naturally regards as impossible.

Other sorcerers were put to death for raising tempests, and some of them, at the stake, confessed their crimes, and rejoiced that they were about to be delivered from the control of their demon lovers.

The inquisitors, who judged these cases, aver that demon-worship had become, as it were, hereditary in certain families, and in certain localities; that it was principally noticed in women, and more especially in young women with a great deal of black hair, who are readily seduced by incubi; thus indicating that demonolatria was more frequent in women than in men; that the hereditary character and example have always exerted an injurious influence on the transmission of insanity, and that young women, such as those described by the inquisitors, are very liable to uterine sensations, which recall to their recollection during sleep that which passes during sexual intercourse. In some instances, it is probable that weak timid persons, in dread of torture, or overcome by pain, confessed that which they knew to be false. All who were executed as demon-worshippers were not, in all probability, monomaniacs.

The nuns of Cambrai, in 1491, were seized with demonopathy, which continued for four years. They used to run like dogs across the country, spring into the air like birds, climb trees like cats, hang on the branches, imitate the cries of animals, divine hidden things, and prophesy the future. At last the demon confessed that he was the author of these extraordinary events, aided by a nun named Jeanne Pothière, with whom he had illicit commerce. Jeanne was condemned to perpetual imprisonment. The recovery of the other nuns took place very slowly. The authors who mention the confession made by the devil, do not state whether it was by the mouth of Jeanne herself, or by one or more of the possessed. In the former case, Jeanne must have been the victim of a perversion in her sensations and intellectual faculties; in the latter, she was the victim of charges invented by persons partially deprived of reason and judgment.

The disease with which the nuns were afflicted, appears to us to be in some way connected with hysterical chorea, tinctured, more or less, with the prevalent superstitions of the time. This is the disease which we fancy M. Calmeil would call hystero-demonopathy.

The sixteenth century is remarkable for the commencement of the struggle of science against superstition, as far, at all events, as regards the diseases of the mind. On the one hand, Barthelèmi de Lépine, de Fernel, Ambrose Paré, de Bodin, de Leloyer, and de Boguet ranged themselves on the side of the theologians, and attributed certain of these diseases, such as demonomania, hystero-demonopathy, zoanthropy, and possession, to commerce with supernatural beings, while, on the other hand, Ponzinibius, D'Alciat, Wier, de Pigray, Jean Baptiste Porta, and Montaigne, with a degree of intelligence and courage which does them immortal honour, removed the veil that obscured the singular phenomena attendant on these forms of mental disease, demonstrated that they were the result of a pathological condition of the mind, and that society was acting with great cruelty in coolly permitting the punishment of numerous maniacs, many of whom were capable of being restored to reason.

De Lépine, who was a professor of theology and a Dominican, was a stout advocate on the side of the theologians. He contended for the truth in every particular of the confessions made by the accused, and averred his belief that they had the power, with the aid of the demon, of changing themselves into cats, a form they generally assumed when about to destroy infants at the breast. The pomade of sorcerers, stated recently by Mr. Huttmann, to be a preparation of red ants, and in some degree allied to chloroform in its action, he asserts had even power over the faithful, while it stupified sorcerers and threw them into a trance. He further contended, that although certain of the sorcerers, after using the ointment, instead of really repairing to the Sabbat, remained torpid, and, as it were, dead in their beds, or in a corner of their house, they ought not, therefore, to be looked upon as innocent, inasmuch as they experienced the same sensations as if they had been present, which they would not have done had they not been engaged in bonds of connexion with Satan, and he further gave it as his opinion that even those who merely experienced illusory sensations and ideas should be put to death, for those who abhor the devil and his works are not subject to such aberrations of the senses and judgment.

Unfortunately these monstrous opinions were rather generally adopted by the demonographers of all countries, and blood flowed plentifully in consequence.

François Pic de Mirandola has no doubt of the cohabitation of fallen spirits with men and women, and he seriously narrates the cases of two priests, who for years maintained such an intercourse. Savonarola, according to him, was frequently visited by spirits, and on one occasion by the Holy Ghost, in the form of a bird with most beautiful plumage. Among those who wrote in favour of the visitation of supernatural beings to this world, and who believed that they saw and conversed with them, are Melancthon, Luther, and Jerome Cardan. Lange narrates a case, as illustrative of supernatural diseases, of a maniac, who slew himself in 1539. When the body was examined after death, there were found in the stomach, a large piece of wood, four knives, two plates of iron, and a mass of hair—all of which, in Lange's opinion, were placed there by diabolical agency. In this opinion, Ambrose Paré agreed.

Fernel, who possessed some notions, drawn from the writings of the ancient physicians, on frenzy, epilepsy, mania, hypochondriasis, and melancholy, of which he admitted several species, believed in the influence of evil spirits on the human body. The state of possession resembled that of ordinary insanity, except that the possessed could read in the past events, and divine the most secret things. They also trembled when they heard the praises of the Creator. Ambrose Paré, the father of French surgery, and Bodin, the juris-consult, fully coincided in all the doctrines of the theologians. The latter, later in life, was himself accused of sacrificing to Beelzebub.

Bodin published a work on demonomania, which, like that of Lépine, tended greatly to prevent the progress of science, and confirm the public belief in the doctrines of the theologians. He apparently gave full credence to all the monstrous tales recorded in the justiciary records of the inquisitors, and states that many females were violated by the fiend the first time they attended the Sabbat. The intercourse of men with incubi, he adds, really occurred, but less frequently. Insanity and somnambulism were occasionally, but not necessarily, induced by supernatural influence. Demoniacs threw up by the mouth pieces of wood, pins, &c., and were subject to strange contortions; in one case, the chin was turned towards the nape of the neck, and the tongue thrust out of the mouth. The devil can speak, by the mouths of the possessed, in languages which they did not understand, and of matters with which they were unacquainted. The demons were drawn into the organism by the sorcerers; they were expelled by exorcisms from the bodies of men and animals, and houses were also purified by the same process, which was not always without danger, as the demon might transfer his residence to the body of the exorcist. St. Gregory and Nider both mention instances of this. The soul, according to Bodin, could be separated from the body for an instant—a phenomenon which really occurs, in his opinion, during ecstasy, and is due to the influence of supernatural beings. The ecstasy of demoniacs was regarded as evidence of their being under Satan's yoke; during its continuance, the soul could make long journeys; and seven persons, who were condemned and burnt at Nantes in 1549, who were in a state of ecstatic immobility for several hours, reported afterwards that they knew all that had passed in the interior and environs of the city during their trance.

Lycanthropia was regarded by many medical men, among whom was Paulus Ægineta, as a species of insanity, in which the maniacs thought they had become wolves, and ran about the woods; Theophrastus, however, Pomponaceus, and even Fernel, were of opinion that lycanthropia was a true and indisputable disease.

Four years after Bodin's work on *Demonomania* was published, there appeared a monograph on *Spectres*, by Leloyer, in which he seeks to show that the voices and other sounds heard by the hallucinates, the objects they fancy they see, &c., are the production of angels and demons. The latter frequently assumed the character and appearance of the dead, and presented themselves as such to the friends of the departed. He designated different kinds of demons, and assigned to each its respective duties and influences. The union of incubi and succubi with human beings might be effected, but it was never fruitful, nor was the metamorphosis of man into animals ever real, although Satan had the art to make the change appear such to the demoniac. The occurrence of insanity depended on the pre-existence of disease in the organs; the demon caused the entire derangement of the operations of the intellect by taking possession of the affected parts. The soul never left the body before the moment of death. During the ecstasy of the sorcerers, it was so pre-occupied by the continuance of the impressions with which it was assailed, by the vivacity of the images, the representation of which was offered it by the devil, that the patient appeared as if deprived of life. Should he present to the soul the pictures of real life, the sorcerer, when he awoke, might faithfully detail events which had passed at a distance from him. The effect is the same as if the soul had really left the body.

Leloyer, in another part of his work, brought forward the opinions of Hippocrates, Galen, Areteus, Nemesius, and Paulus Ægineta, on hallucinations, melancholy, ecstasy, lycanthropia, mania, and other disorders of the understanding, and acknowledged that all cases of these diseases were not caused by the intervention of evil spirits, and thereby tacitly admitted that it was possible they might all arise from pure derangement of the nervous system. He further stated, that those who fell into ecstasy rubbed themselves first of all with a narcotic ointment; and therefore that the effects attributed to a supernatural cause might result from the toxic agent which had been used.

We come next to writers whose productions must give greater satisfaction. They are those who sought to prove that the alleged cases of sorcery, &c., were in reality instances of disordered intellect.

Ponzinibius, the first on the noble list, wrote that demonolatria was a disease, and all the sensations which caused the Lamias to believe they were demon-worshippers should be attributed to the depraved state of the senses; that it was false, that certain persons could assemble at night, unknown to their families, in places frequented by spirits; the crimes of which they were accused could not be proved legally, and further, that it was atrocious to burn maniacs. The council of Ancyra declared that the abominations attributed to the sorcerers could not be committed in reality. The inverisimilitude of the events described by the alleged demoniacs ought to have opened the eyes of all reasonable people; nor could he comprehend how they could admit as facts things which were in opposition to the laws of nature.

Alciat followed, and feared not to attack an inquisitor, who had put a number of hallucinates to death in Piedmont. He supported his views by the declaration of the council of Angouri, by whom the absurdity of the sorcerer's sabbat was declared. He brought forward the evidence of the husbands of the alleged sorceresses, who asserted that their wives had not left their beds, nor been out of their presence, and attributed the assertions of the demoniacs to melancholia, and believed they could be cured, if their state of penury permitted the requisite attention. Nor did he allow that the occurrence of ecstasy was a reason for depriving its victims of life, as it at once proved that the alleged crimes could not really have been committed.

Lemnius Levinius explained the strange sounds sometimes uttered by the possessed, by the intensity of the cerebral excitement, and not as in any way induced by demons. It was occasionally cured with great rapidity by therapeutic measures. Epilepsy, which had also been attributed to supernatural influence, had not any such origin, which should be sought in the encephalon and the humours.

Wier appears to have been well acquainted with the spirits; he calculated their numbers as several millions, and admitted that they possessed real influence in the productions of the conditions which have been hitherto under consideration. He, however, did good service by tracing the causes which lead to insanity. He has shown that the maniacs of the sixteenth century often attempted suicide; that they frequently swallowed pieces of bone, feathers, and iron; that convulsions, complicated with delirium, frequently occurred among them, and in boys' schools, and sometimes were epidemic; that the delirium of demonolatria assumed the most diverse forms; that persons were discovered who simulated demonopathy, and that many toxic agents were known which, when taken into the stomach, could cause a momentary delirium. He meditated deeply on the symptoms of demonomania, lycanthropia, and religious melancholy; he examined many maniacs, reflected on the processes against sorcery already instituted, and was convinced that lycanthropes (wehr-wolves) and stryges, whom the theologians were so anxious to burn, were no other than maniacs. He appeared to have believed, however, that Satan led them to make the false statements which they promulgated, but without any culpability on their part. The children they pretended to have devoured were still living; the dead they asserted they had torn from the tomb, were still in their graves. The sorcerers could be bound down with chains in their beds; they nevertheless declared that they had danced at the Sabbat, and had connexion with incubi. Those who, they stated, had been present at the Sabbat, were seen elsewhere at the very time by witnesses of credibility. If they did commit crimes, it was because they were no longer able to appreciate the bearing of their nature, nor to control their own impulses. He was, further, of opinion that the homicidal monomania of the Vaudois could be credited only by imbecile or ignorant persons; and that the stryges, whose blood was so freely shed on the banks of the Leman and Rhine, in Savoy, and elsewhere, had neither murders nor other crimes to reproach themselves with. He added, further, that the confessions were obtained by throwing old persons, whose intellects were already disordered, into dark, cold, and damp dungeons, where grief, despair, and fright, combined with the torture and the effects of the soporifics given them by brutal judges, completed the work, and ruined the intellectual and moral powers.

Numerous cases were reported to have occurred in this century of fearful suicides and homicides, in the majority of which the writers who have published the details appear to recognise indications of insanity; nor was it long afterwards before the fact was recognised that insanity frequently becomes homicidal. Thus was a step made in the right path. About the middle of the same century, a villager near Pavia, says Fincel, thought himself changed to a wolf, and killed several persons. When taken, he still asserted that he was a wolf, and differed from them only in this, that in ordinary wolves the hairy coat was outside, while in his it was between the skin and flesh. Some persons, desirous of ascertaining this, made incisions in his arms and legs, and then recognising the innocence of the maniac, called in surgical aid. He died, however, a few days afterwards. Fincel added that he classed certain lycanthropes among the insane. Towards the close of the century, writers began more generally to attribute these and such like diseases to aberration of the mind. Instances will be found in the works of Valeriola, Zuynger, Brassavola, Dodoens, Donat, Altomare, Schenck, and Houlier, which at an earlier period would have been regarded as genuine cases of sorcery.

In 1580, Nicholas Lepois published a treatise on pathology, no less remarkable for the solidity of its doctrines than for its freedom from the ruling prejudices. Catalepsy, frenzy, coma, amnesia, palsy, apoplexy, incubus, convulsions, mania, melancholy, epilepsy, are all referred by him to so many lesions or derangements of the encephalon, and described with much talent. He was not content with localizing, as much as possible, the *point de départ* of the morbid phenomena, and comparing them together in the different nervous affections, in order to ascertain the differential diagnosis, he also sought to appreciate their nature and severity, and to make known the means requisite in each case to restore the functional equilibrium. He however did not feel himself justified in denying the existence of demoniac insanity.

The writings of wise and learned men, such as those already cited, together with the essays of Montaigne, in which, with great talent, he combated the opinions and practice then in vogue with respect to sorcerers, had little or no effect on the clergy, who held the scales of ecclesiastical justice, and on the lower orders of society, who, throughout Europe, lay under the yoke of the most deplorable ignorance. The belief in all the horrible tales which we have noticed was prevalent through the Christian states; it became, as it were, impossible to diagnose between murderers and hallucinates or lypemaniacs. Monks assumed the office of physicians, and endeavoured to cure the insane by exorcisms. Demonomaniacs were led to the scaffold in bands of dozens, and even of hundreds. One hundred thousand were put to death in the name of justice during the reign of Francis the First.

Demonomania appears to have been the principal crime for which these unhappy victims suffered in 1507; thirty women were burnt by the inquisition at Caluhorra for sorcery. From 1504 to 1523 we are told that sorcery prevailed epidemically throughout Lombardy, and several popes launched their bulls against the offenders. The Dominicans, according to Lepine, burnt a thousand wretches, principally women, annually at Come. The details of the supposed crime resemble in their general aspect those already narrated.

demon-worship, homicide, infanticide, and zoanthropia constitute the principal features. In 1521 two men were burnt at Poligny for demonomania, lycanthropia, and homicide. They confessed five murders, committed while disguised as wolves, and acknowledged to have had connexion with she-wolves. Their victims, according to their account, were chiefly of the female sex, and were generally devoured by them after they had been murdered. These statements M. Calmeil justly regards as hallucinations, and he cites a case, narrated by Guillaume D'Auvergne, of another lycanthrope, who boasted of his deeds as a wehr-wolf, but who, when followed, was found to retire to a solitary cave, and pass his time in ecstasy, after which he again recounted his feats of lycanthropy, and boasted of the terror he had caused in the neighbouring villages.

One hundred and fifty women were sentenced in Navarre to imprisonment and flogging on their confession of demonolatria, and a number more were burnt at the stake in Saragossa by order of the inquisition.

In 1574 Gilles Garnier, known as the hermit of St. Bonnot, was burnt for lycanthropy and numerous homicides. There can be but little doubt of his having murdered and eaten several children, and even attacked horsemen on the public highway, as he was, on more than one occasion, disturbed and put to flight at the moment of destroying his victims. His insanity is equally evident. In the same year eighty demonomaniacs were burnt alive in Savoy, and three years afterwards nearly four hundred in Languedoc. In 1578, Jeanne Hervilliers, whose mother was burnt for sorcery, was also condemned to the stake for similar offences. Homicide and carnal intercourse with the demon she freely confessed. Here, as in other cases, the hallucinations of the maniac led her as a criminal to the stake. Pigray, surgeon to Henry the Fourth, was with others appointed in 1589 to examine fourteen demonomaniacs, who had been condemned to death, and after having ascertained the phases of their malady, reported that "a dose of hellebore would be more serviceable than punishment." The court dismissed the accused accordingly. Seven years prior to this decision, a number of demoniacs were burnt to death by the inquisition at Avignon on a charge of demonolatria, which was generally confessed by the unfortunate victims, in whom hallucinations had apparently been produced by extreme famine, so severe, indeed, that they had been obliged to live on the egesta of horses and asses, and on wild plants which they were enabled to gather—a cause capable in all ages to produce insanity. In Lorraine, nine hundred victims were burnt in fifteen years, and many others were driven to commit suicide.

Voltaire states that, between 1598 and 1600, more than six hundred lycanthropes or demoniacs were put to death in the district of the Jura by Boguet. This he avers on the dictum of the judge himself. The statements made by the accused were of a most extraordinary character in every respect, and such as no person in possession of common sense could consider as other than the ravings of insanity. While these events were passing on the Jura, a man was arrested at Angers on a charge of lycanthropy and homicide, he having been found near the mangled body of a boy, with the inside of his hands bloody. Two wolves made their escape just as the rescuers arrived. In his confession, he acknowledged that he had killed the boy, and eaten part of him, and the two wolves who got away were his brother and cousin. The judgment, of course, was a sentence of death; but an appeal was made to the parliament of Paris, which wisely decided that the case evinced more insanity than sorcery, and ordered his confinement in a lunatic asylum. In the same year, 1598, the priest of Payas was burnt in the Limousin on a charge of demon-worship, which he strenuously denied, until obliged to confess by the application of torture.

During the sixteenth century, hystero-demonopathy became endemic in several places, more especially in convents and schools. It broke out about 1550, and lasted till 1565. The disease is described in old books as the "possession *des nonnains*," and the account given of it shows that almost all the encephalic functions were more or less simultaneously affected in the persons who were supposed to be possessed. It first showed itself in the convent of Uvertet, in Hoorn, immediately after Lent, consequently just after the nuns had endured considerable privations for more than forty days. It lasted among the nuns above three years, and then began to diminish in intensity. Unfortunately, during this time, several persons were arrested charged with causing the disease, and one poor woman was tortured to death. The disease then broke out among the nuns of St. Brigitte, the first person attacked being a young woman who had taken the vows from a disappointment in love; from her it spread by contagion, or, rather, by imitation, among the others. It continued about ten years. The nuns in the convent of Neomage were affected with hallucinations of the organs of hearing, and fancied they heard musical instruments at night-time in the dormitories. These imaginary sounds were attributed to the demon endeavouring to seduce the sisters. The nuns of Kintorp, near Strasburgh, were affected in 1552. At first, a few only were attacked; the disease afterwards invaded others by contagion. The attack was violently convulsive, the muscles of the pharynx participating. As soon as it was known that one of the nuns was thus affected, all those who slept in the same dormitory were also seized. The return of the fits was always preceded by fœtid breath. They all complained of a burning sensation on the soles of the feet, as if boiling water had been poured on them. The unfortunate cook of the establishment, although subject to the same disease as the nuns, was denounced as being in communication with the fiend, and as the cause of all the mischief; her attacks were said to be simulated. Herself and another were burnt to death, but this tragical event did not arrest the progress of the demonopathy, which spread among the neighbouring villages, many other persons being put to death as sorcerers on that account. In 1554, the same disease showed itself among the converted Jewesses, at Rome. Those attacked were above eighty in number, and had just been baptized. The Jews were accused of having bewitched them, and would, in all probability, have fearfully expiated the charge, but that a Jesuit boldly and honestly defended them before the pope, and showed that men do not possess the power of sending demons into the bodies of their fellow-creatures. It would have been well if other theologians had possessed the good sense of this Jesuit. Ten years afterwards the nuns of Nazareth, at Cologne, were similarly affected, and in addition, laboured under nymphomania, — in this instance, at least, clearly showing the origin of the disease in a disordered condition of the uterine organs. A convulsive disease broke out in the orphan establishment at Amsterdam, in 1566, thirty children, according to Wier, seventy, according to Neal, being affected. The greater number of these were boys.

The case of Dr. Torralba, and that of the old abbess of Cordova, which occurred in this century, are of decided interest; but we fear the extent of this article will prevent our giving them the extended notice which they deserve. Dr. Torralba was a very learned physician, who had travelled over great part of Europe to increase his store of knowledge. After a time his character changed; he became sullen, and although hitherto firm in his religious and philosophical belief, he became tormented with painful doubts, and gave himself up to chiromancy. The insanity with which Torralba was afflicted gradually increased, and he began to fancy that he was attended by agencies, who carried him about from place to place with great rapidity, and enabled him to prophecy. His genius was exceedingly learned, and could converse with him in all languages. He was, however, unable to exhibit him to others, who earnestly desired to see him. In 1528, Torralba was arrested by the inquisition, and tortured, with the view of discovering whether his familiar were a good or an evil spirit. The latter he denied; and asserted that he had not made any compact with him. After three years' uncertainty, and physical and moral sufferings, Torralba was condemned to abjure his errors as a heretic, to renounce the demon, to wear the *san benito* publicly, and to be imprisoned for a certain time. His life was only spared at the instance of the court, and of some friendly grandees. The abbess of Cordova had enjoyed all her lifetime, till old age, the reputation of holiness, and of working miracles. Labouring, then, under a disease which it was considered would prove fatal, and in terror for her future welfare, she confessed her intercourse with demons, which at first represented themselves to her as angels, and even as the Christ, and by their aid she said she had effected all her miracles. She was condemned to do penance publicly, and to be shut up in a convent for the remainder of her existence. Both cases were instances of monomania.

